# Virus Like Particles (VLP) as multivalent vaccine candidate against Chikungunya, Japanese Encephalitis, Yellow Fever and Zika Virus

**DOI:** 10.1038/s41598-020-61103-1

**Published:** 2020-03-04

**Authors:** Himanshu Garg, Tugba Mehmetoglu-Gurbuz, Anjali Joshi

**Affiliations:** Center of Emphasis in Infectious Diseases, Department of Molecular and Translational Medicine, Texas Tech University Health Sciences Center, El Paso, TX USA

**Keywords:** Protein vaccines, Viral infection

## Abstract

Mosquito borne viral diseases are an emerging threat as evident from the recent outbreak of Zika virus (ZIKV) as well as repeated outbreaks of Chikungunya (CHIKV), Yellow fever (YFV) and Japanese encephalitis (JEV) virus in different geographical regions. These four arboviruses are endemic in overlapping regions due to the co-prevalence of the transmitting mosquito vector species *Aedes* and *Culex*. Thus, a multivalent vaccine that targets all four viruses would be of benefit to regions of the world where these diseases are endemic. We developed a potential Virus Like Particle (VLP) based multivalent vaccine candidate to target these diseases by using stable cell lines that continuously secrete VLPs in the culture supernatants. Moreover, inclusion of Capsid in the VLPs provides an additional viral protein leading to an enhanced immune response as evident from our previous studies with ZIKV. Immunization of Balb/c mice with different combinations of Capsid protein containing VLPs either as monovalent, bivalent or tetravalent formulation resulted in generation of high levels of neutralizing antibodies. Interestingly, the potential tetravalent VLP vaccine candidate provided strong neutralizing antibody titers against all four viruses. The 293 T stable cell lines secreting VLPs were adapted to grow in suspension cultures to facilitate vaccine scale up. Our stable cell lines secreting individual VLPs provide a flexible yet scalable platform conveniently adaptable to different geographical regions as per the need. Further studies in appropriate animal models will be needed to define the efficacy of the multivalent vaccine candidate to protect against lethal virus challenge.

## Introduction

Arthropod borne viruses are a group of pathogens that are transmitted in the human population via the bite of arthropods including mosquitoes, flies and ticks. Recent increase in arboviral epidemics in the human population has been attributed to various factors including urbanization and geographical expansion of both the host and vectors for these diseases^1,2^. Amongst these arboviral diseases, Chikungunya (CHIKV), Japanese Encephalitis (JEV), Yellow Fever (YFV) and Zika virus (ZIKV) have expanded from isolated outbreaks in endemic areas to large scale epidemics affecting multiple continents^3,4^. The recent outbreaks of ZIKV^5^ and previous outbreaks of CHIKV^6^ are indications that these viruses will continue to plague the human population putting millions at risk and adding a significant burden on the healthcare system.

The World Health Organization lists Dengue virus (DENV) as the most common mosquito-borne viral disease with CHIKV, YFV and JEV as other arboviruses being endemic in various parts of the world. The majority of human population that resides in the tropical and subtropical regions of the world is at risk for at least one of these arboviral infections. These pathogens are transmitted via vectors that are found in overlapping geographical regions; *Aedes sp* in the case of CHIKV, YFV and ZIKV and *Culex sp* in the case of JEV^2^. Furthermore, the clinical symptoms of infection with these viruses can be remarkably similar making differential diagnosis difficult^7^. To complicate matters further, the serum cross reactivity between these viruses makes differential diagnosis and sero- surveillance a daunting task^8^. Hence, an effective control of these viruses requires active vaccination in endemic areas and in the most vulnerable populations. Currently, a live attenuated virus (LAV) vaccine for YFV and a LAV as well as a killed JEV vaccine is available for use in humans^9,10^. However, these vaccines utilize viral strains from the early 70 s and have limitations including safety and availability. Similarly, a tetravalent LAV vaccine for Dengue has recently been approved with limited success and much controversy regarding its effectiveness^11^.

While LAV vaccines have been the candidate of choice in the past, recent advances in molecular biology has demonstrated the effectiveness of other vaccine platforms especially Virus Like Particles (VLPs). The success of the multivalent Human Papilloma Virus (HPV) vaccine has demonstrated the safety and efficacy of VLPs as vaccines^12^. Previously, we developed a VLP vaccine candidate for Zika virus and found that the immunogenicity of Capsid protein containing VLPs (CprME) was better than prME VLPs^13^. Furthermore, the use of stable cell lines to generate VLPs provides a much needed platform for the advancement and commercialization of this technology^14^. Besides being safe and efficacious, VLP vaccines also provide the added advantage of multivalency by combining closely related different VLPs in desired concentrations into a single formulation^15^. The HPV vaccine is one such example where VLPs from 9 closely related strains of HPV are included to increase antigenic breadth and generate a broader immune response^16^.

In this study, we used the VLP approach to develop a safe and efficacious vaccine targeting CHIKV, JEV, YFV and ZIKV termed (CJaYZ vaccine). Stable cell lines secreting Capsid protein containing VLPs for all four viruses were established and characterized. Concurrently, Reporter Virus Particle (RVP) assays were also developed for the four viruses to test neutralizing antibody activity. Immunogenicity of the VLPs as monovalent, bivalent or tetravalent combinations was tested in Balb/c mice. High level of neutralizing antibodies were generated against each virus both in monovalent as well as tetravalent combinations. To the best of our knowledge, this is the first study demonstrating the effectiveness of Capsid protein containing VLPs for YFV and JEV as well as the first report demonstrating the efficacy of a multivalent VLP vaccine candidate against arboviruses. Our study demonstrates the feasibility of a multivalent VLP vaccine to provide neutralizing antibody response against diverse arboviruses.

## Results

### Synthetic gene constructs expressing structural proteins of YFV, CHIKV and JEV and generation of Capsid protein containing VLPs

**Figure 1:**
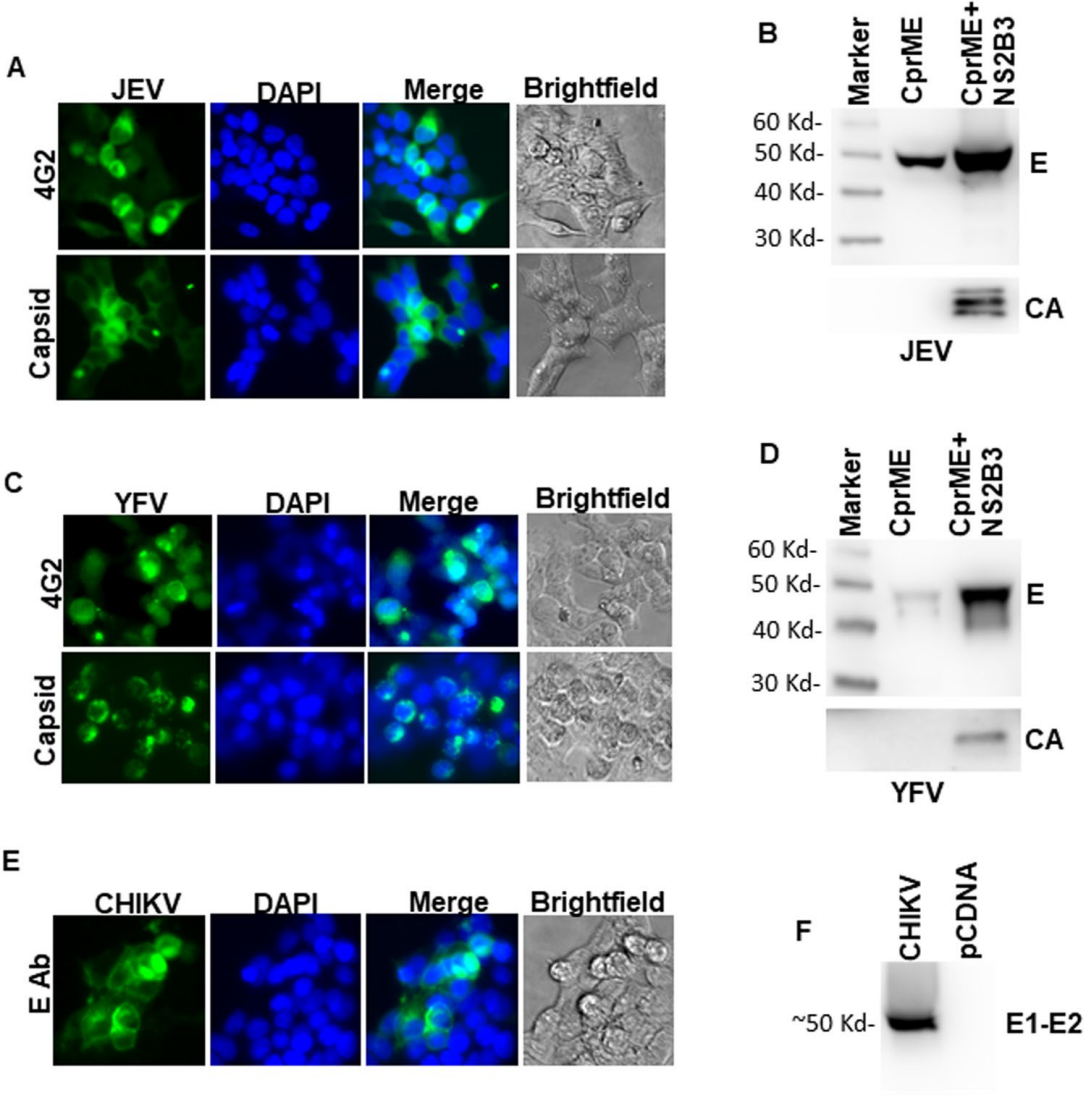
Characterization of JEV, YFV and CHKV protein expression and VLP release. **(A)** 293 T cells were transfected with JEV CprME expression vector. Cells were analyzed for E protein and Capsid protein expression after staining with respective antibodies followed by fluorescence microscopy. **(B)** 293 T cells were transfected with JEV CprME expression vector alone or the JEV CprME vector along with Zika NS2B-3 expression plasmid. Culture supernatants were ultracentrifuged and analyzed for E protein and Capsid protein expression by western blotting. **(C)** 293 T cells were transfected with YFV CprME expression vector and analyzed for E protein and Capsid protein expression by immunofluorescence microscopy. **(D)** 293 T cells were transfected with YFV CprME expression vector alone or the YFV CprME vector along with Zika NS2B-3 expression plasmid. Culture supernatants were ultracentrifuged and analyzed for E protein and Capsid protein expression by western blotting. **(E)** 293 T cells were transfected with CHKV expression vector and analyzed for E1-E2 protein expression by immunofluorescence microscopy. **(F)** 293 T cells were transfected with CHKV expression vector. Culture supernatants were ultracentrifuged and analyzed for E1-E2 protein expression by western blotting. Images A, C and E were analyzed using NIS Elements AR software version 3.2 (Nikon; https://nis-elements-viewer.software.informer.com/3.2/). Images B, D and F were analyzed using GENETOOLS gel analysis Software version 4.03 (f) (Syngene, https://www.syngene.com/software/genetools-automatic-image-analysis/).

To develop a multivalent arboviral vaccine, we first designed structural protein expression vectors for the above viruses. For ZIKV, YFV and JEV it included the CprME genes and for CHIKV the C-E3-E2-E1 genes. We have previously developed a VLP based platform for Zika Virus using a codon optimized synthetic gene construct^13^ and used a similar approach for developing structural protein constructs for the other arboviruses with minor modifications. We developed a consensus sequence for CHIKV, JEV and YFV to include the most representative circulating virus sequences in our vaccine platform. Full length genomic sequences from the ViPR database were used to generate a consensus sequence using DNAStar software. This consensus sequence was then used to generate a codon optimized expression vector spanning the structural proteins of CHIKV, JEV and YFV. Codon optimized sequences were synthesized commercially and cloned into pcDNA 3.1 vector and tested for protein expression. As demonstrated in Fig. 1, CHIKV, JEV and YFV constructs showed E expression upon transfection followed by fluorescence microscopy. For, JEV and YFV both E protein and Capsid protein expression was documented **(**Fig. 1A,C**)** and for CHIKV, robust E protein expression could be detected as well **(**Fig. 1E**)**. ZIKV protein expression using a similar approach has been characterized previously^13,14^.

Having successfully generated the structural protein expression vectors, we next analyzed VLP formation from our codon optimized structural protein expression vectors. The cleavage of capsid from prME regions in flaviviruses is mediated by the NS2B-NS3 protein complex that forms an active protease^17^. The viral protease cleaves off the Capsid protein from the cytoplasmic side of the ER. This is required for the cell signalase to cleave off and release the prM^17–19^. Expressing NS2B3 in trans is one method of providing the protease for cleavage and has been used previously for Zika virus VLP production. We wanted to test whether JEV and YFV CprME would be cleaved by the Zika NS2B3 protease. Cells were cotransfected with ZIKV NS2B3 with either YFV or JEV CprME and the supernatants were assayed for VLP production. As demonstrated in Fig. 1B,D, JEV and YFV constructs showed minimum VLP release into the supernatants in the absence of NS2B3 expression. Protease expression robustly increased VLP secretion as detected by both E and CA antibodies. Moreover, both ZIKV and WNV NS2B3 were efficient in cleaving ZIKV, YFV and JEV CprME with comparable VLP release in the supernatants **(**Supplementary Figure 1**)**. For CHKV, robust VLP secretion was detected via western blotting when probed with E1-E2 antibody **(**Fig. 1F**)**. Thus, our CprME expression constructs show cellular expression of desired proteins and expression of Zika NS2B3 with other flaviviral CprME can produce Capsid protein containing VLPs.

### Reporter Virus Particle based neutralization assay is sensitive and specific

Reporter virus particles represent a specific and sensitive assay to study various aspects of virus biology especially viral entry^20,21^. These pseudovirions are capable of infecting cells once in a manner identical to the native virus but are non-replicating. In case of flaviviruses, the structural proteins (CprME) from a separate plasmid can complement a subgenomic WNV replicon expressing a GFP reporter gene to generate RVPs. This RVP based system has been shown to work well for different flaviviruses including WNV and Zika^13,21^. We generated RVPs expressing JEV and YFV and ZIKV structural proteins and tested them for infectivity in Vero cells. As shown in Supplementary Figure 2A–C, Vero cells showed infection by JEV, YFV and ZIKV RVPs in a dose dependent manner.

For CHIKV we used a slightly different approach as it is an alpha virus. Previously, Akahata *et al*.^22^, demonstrated that HIV reporter particles pseudotyped with CHIKV Env generate a specific and sensitive RVP system that can be used for neutralizing antibody detection. To develop CHIKV RVPs, pcDNA3.1 vector expressing the Env region was co-transfected with HIV NL.LucR-E- vector to generate luciferase expressing CHIKV pseudotyped RVPs. Once again, infection of Vero cells in the presence of serial dilutions of CHIKV RVPs showed a dose dependent luciferase signal **(**Supplementary Fig. 2D**)**.

**Figure 2:**
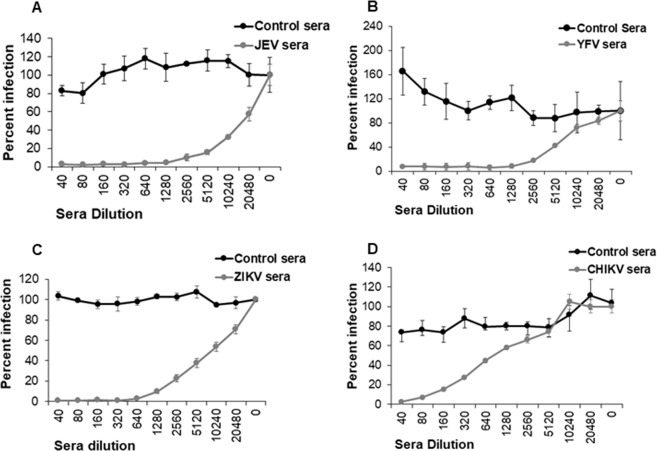
Neutralization of RVP infection using disease specific sera. Vero cells were infected with **(A)** JEV RVPs **(B)** YFV RVPs **(C)** ZIKV RVPs or **(D)** CHIKV RVPs after incubation with serial dilutions of virus specific sera or control sera. Infection was determined 72hrs post infection via automated microscopy. Data is represented as percent infection normalized to control sera as mean ± SD of triplicate observations.

Having developed RVP based infectivity assays for Zika, JEV, YFV and CHKV, we next asked if infection via RVPs was Env specific and if it could be inhibited via specific sera. As demonstrated in Fig. 2A–D there was good neutralization of individual RVPs in the presence of specific sera in a dose dependent manner. Moreover, no inhibition of JEV, YFV, ZIKV and CHIKV RVPs was seen in the presence of matched control sera. These data demonstrate that the RVP based assay is highly sensitive and specific and can be effectively used for detection of a neutralizing antibody response.

### Bicistronic vector expressing flaviviral CprME and Zika NS2B3 produces Capsid protein containing VLPs

**Figure 3:**
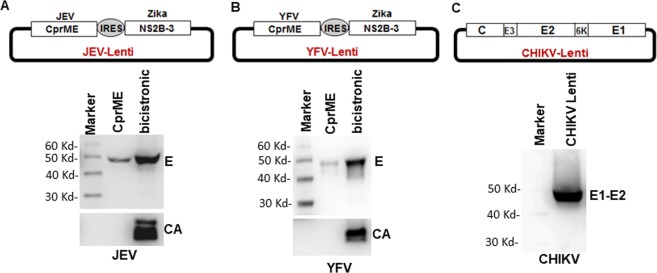
Generation and characterization of bicistronic lentiviral vectors expressing flaviviral structural proteins. **(A)** JEV CprME was cloned into a lentiviral vector that included an IRES sequence followed by the Zika NS2B-3 protease. 293 T cells were transfected with the JEV CprME construct alone or the bicistronic JEV lentiviral construct. Culture supernatants were harvested and VLPs analyzed for JEV E protein and Capsid protein secretion via western blotting. **(B)** YFV CprME was cloned into a lentiviral vector as above and VLP secretion determined in the culture supernatants by western blotting for E and Capsid protein. **(C)** CHIKV C-E3-E2-E1 genes were cloned into a lentiviral vector and VLP secretion determined in the culture supernatants by western blotting for E1-E2 protein. Images were analyzed using GENETOOLS gel analysis Software version 4.03 (f). (Syngene, https://www.syngene.com/software/genetools-automatic-image-analysis/).

The presence of Capsid protein in a flaviviral vaccine can provide an additional immunogen and higher neutralizing antibody titers as seen in our Zika VLP vaccine studies^13,14^. In order to produce CprME VLPs, NS2B3 expression can be achieved either in trans as in Fig. 1 above or from a bicistronic vector. The use of lentiviral vectors to stably transduce cells provides a much needed advantage for generation of VLP secreting cell lines. We have previously developed a biscistronic vector that co-expresses ZIKV CprME and NS2B3 using an IRES sequence between the ORFs^14^. We used the same vector to replace Zika CprME with either JEV or YFV CprME and tested them for VLP production. As demonstrated in Fig. 3, the bicistronic vectors resulted in robust production of JEV and YFV VLPs in the supernatant that show incorporation of Capsid protein. Moreover, the CHKV lentiviral vector also showed robust VLP secretion in the culture supernatants. Thus, a bicistronic vector expressing flaviviral CprME and Zika NS2B3 robustly secretes Capsid protein containing VLPs into the supernatant.

### Generation of stable cell lines producing ZIKV, YFV, JEV and CHIKV VLPs

**Figure 4:**
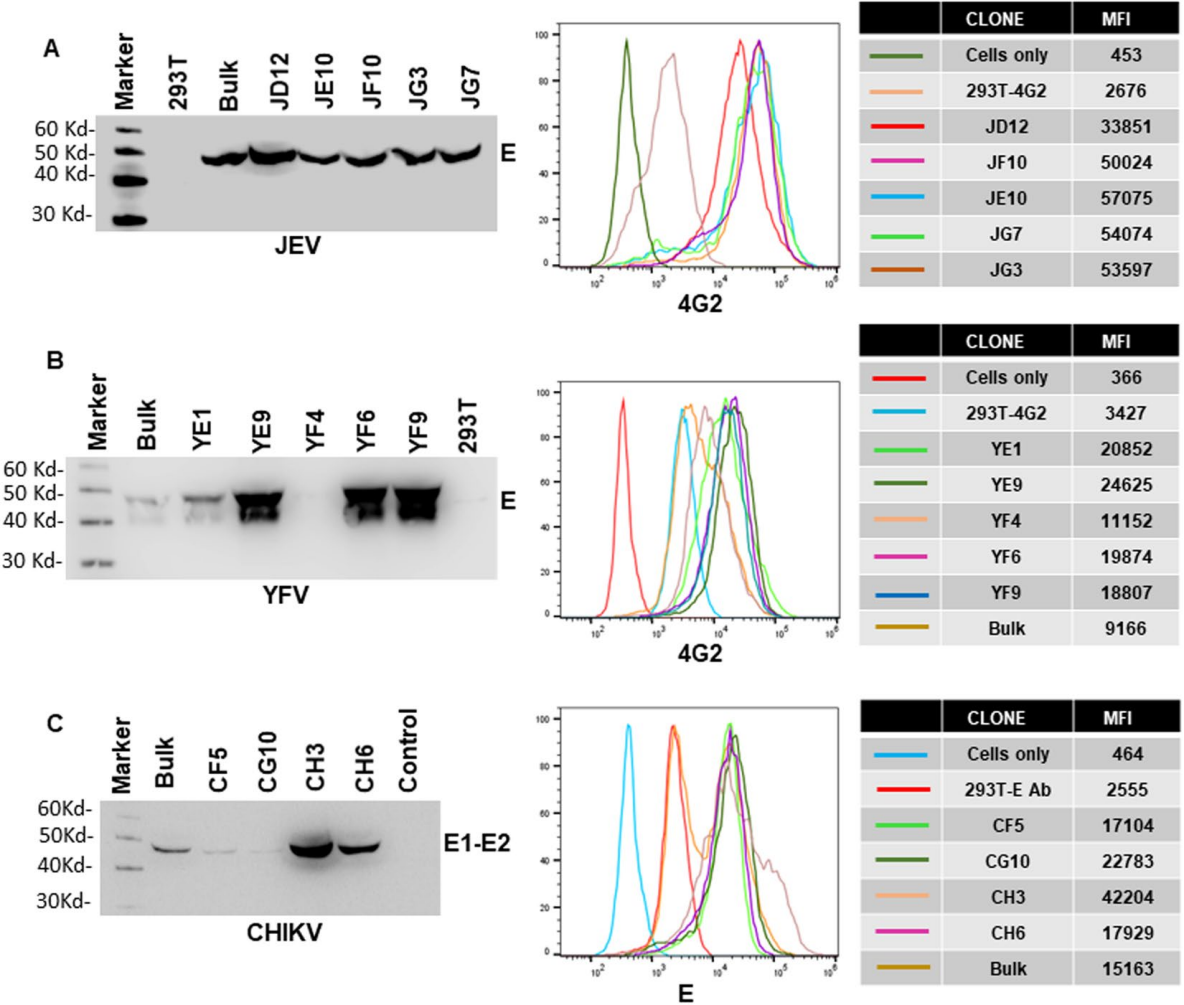
Generation of stable cell lines secreting JEV, YFV and CHIKV VLPs. 293 T cells were transduced with **(A)** JEV **(B)** YFV or **(C)** CHKV structural protein containing lentivirus particles. Transduced cells were bulk selected with blasticidin followed by limiting dilution single cell cloning. For each virus, several single cell clones were characterized for Envelope protein staining on the cell surface via flow cytometry and VLP secretion into the culture supernatants via western blotting. Mean Fluorescence intensity (MFI) of Envelope protein staining for the different single cell clones for JEV, YFV and CHIKV is shown on the right. Gel images were analyzed using GENETOOLS gel analysis Software version 4.03 (f) (Syngene, https://www.syngene.com/software/genetools-automatic-image-analysis/). Flow cytometry histograms were created using the FloJo Software version 10.6.0 (Tree Star, https://www.flowjo.com/solutions/flowjo/downloads).

Several successful VLP vaccines are currently in clinical use suggesting that this approach is commercially viable^23^. Development of stable cell lines secreting VLPs provides an added advantage as the vaccine can be readily scaled up for commercial production. Using the bicistronic lentivirus vector expressing Zika NS2B3 and flaviviral CprME’s, we generated stable cell lines that secrete high levels of JEV, YFV **(**Fig. 4A,B**)** and ZIKV VLPs^14^. For CHIKV, the lentivirus construct expressed the CE3E2E1 region. Lentivirus particles were used to transduce 293 T cells and transduced cells selected by culturing in the presence of Blasticidin. Single cell clones were characterized individually by analyzing E protein expression by flow cytometry and VLP secretion into the culture supernatants. As demonstrated in Fig. 4A, for JEV, the stable cell clone JD12 was best in VLP secretion and showed uniform E protein staining by flow cytometry. Similarly, the YF9 clone was selected for YFV **(**Fig. 4B**)** and CH3 clone for CHKV **(**Fig. 4C**)** for future analysis. The ZIKV E2 cell line has been described and characterized before^14^. Using this strategy we were able to find high VLP secreting single cell clones for JEV, YFV, ZIKV and CHIKV. These cell clones were used for VLP generation for immunization studies in mice.

### Testing stability of single cell clones producing various VLPs

**Figure 5:**
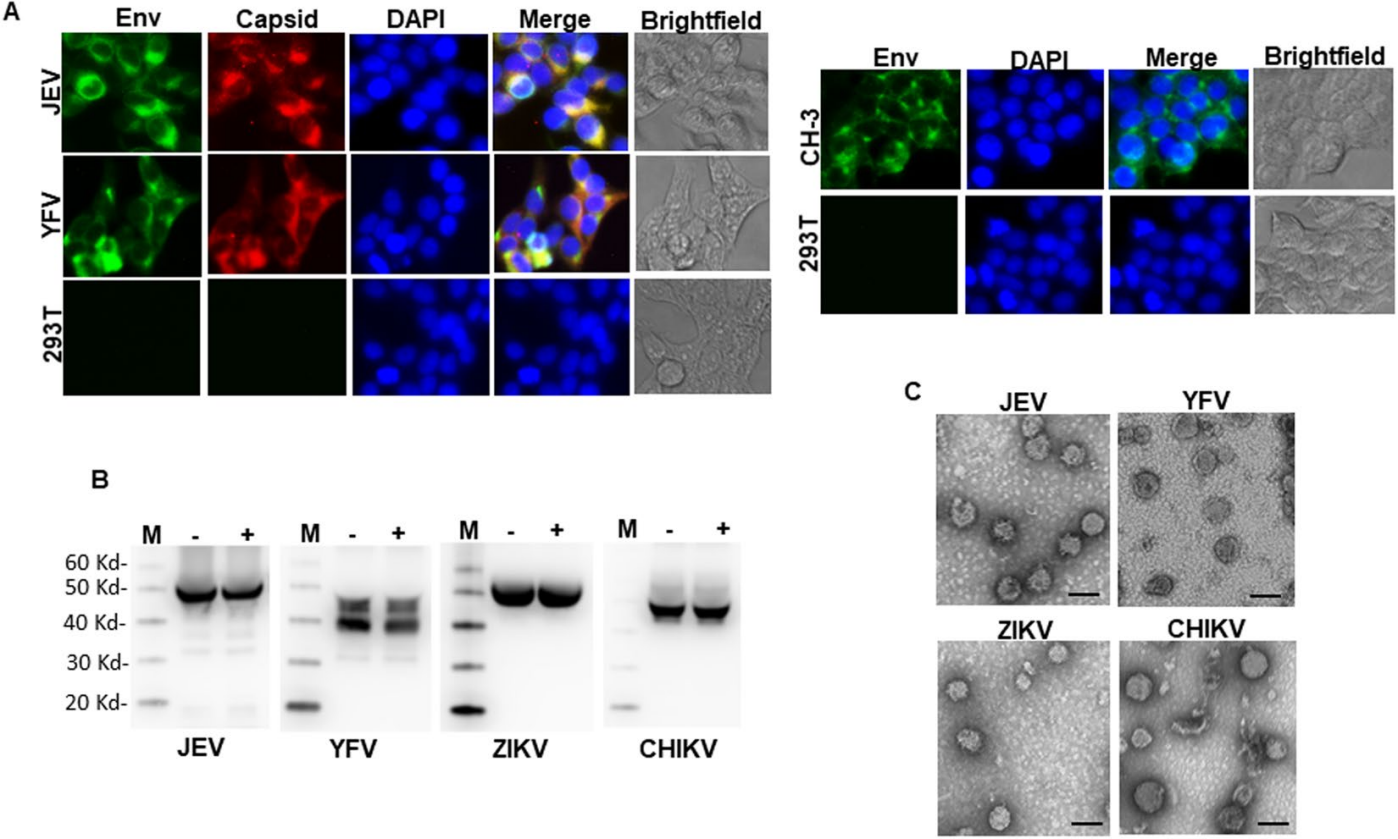
Characterization of stable single cell clones. **(A)** JEV-JD12, YFV-YF9 and CHIKV-CH3 cell lines were stained for E and/or Capsid protein expression. Cells were analyzed by fluorescence microscopy and images acquired. **(B)** The stable cell lines were cultured in the presence (+) or absence (−) of blasticidin for a period of 30 days with routine sub-culturing. Culture supernatants were analyzed for E protein expression by western blotting. M = Protein molecular weight marker. **(C)** VLPs harvested from the individual stable cell lines were negatively stained and analyzed by Transmission Electron Microscopy. Scale = 50 nm. Fluorescence microscopy images were analyzed using NIS Elements AR software version 3.2 (Nikon; https://nis-elements-viewer.software.informer.com/3.2/). Gel images were analyzed using GENETOOLS gel analysis Software version 4.03 (f) (Syngene, https://www.syngene.com/software/genetools-automatic-image-analysis/).

For a vaccine candidate to be successful, it is desirable that there is minimum variability between preps and the cell line is stable and does not lose expression of the exogenous gene. Large scale expansion will also require the growth of cells in media free from selection agents like Blasticidin. To satisfy the above criteria, we tested the stability of the cell lines by two separate methods. The cells were cultured in the presence of Blasticidin for ~9 months. Thereafter, the cells were stained for E and Capsid protein expression and analyzed by fluorescence microscopy. As demonstrated in Fig. 5A, the stable cell lines showed robust expression of E and Capsid proteins despite long term culture, suggesting stable integration of the exogenous gene. We next asked whether the cell lines were capable of robust VLPs secretion in the absence of blasticidin which may be desirable during bulk vaccine preparation. For this the cell lines were cultured in the absence of blasticidin for 30 days. After the terminal passage and when cells reached confluency, culture supernatants were concentrated and analyzed for VLP secretion via western blotting. Cells cultured alongside in the presence of Blasticidin were used for comparison. As demonstrated in Fig. 5B, cells cultured in the absence of Blasticidin showed comparable VLP production for all four cell lines (JEV, CHIKV, YFV and ZIKV) as cells cultured in the presence of Blasticidin. We next analyzed the morphology of VLPs produced from the stable cell lines after long term culture. The morphology of JEV, CHIKV, YFV and ZIKV particles was consistent with that of virus particles with an average diameter of 40–50 nm or slightly larger for CHIKV and densely staining membranes as demonstrated by electron microscopy **(**Fig. 5C**)**. These data demonstrate that the generated single cell clones show stable incorporation of the exogenous gene over a prolonged period of time and show robust VLP secretion in the absence of Blasticidin.

### Immunization of mice with monovalent, bivalent or tetravalent (CJaYZ) arboviral vaccine induces high levels of neutralizing antibodies

Having developed a robust system to produce VLPs, we next proceeded with testing the immunogenicity of the multivalent vaccine in murine model. As the vaccine is produced as individual VLPs **(**Supplementary Fig. 3A**)**, it is important to test the monovalent formulation along with bivalent or tetravalent combinations. We included a bivalent formulation that included JEV and CHIKV VLPs as these two viruses are prevalent in Asia and a second bivalent group consisting of YFV and CHIKV VLPs as these two viruses are endemic in South America and Africa^1^. Balb/c mice in groups of 6 each were immunized with either monovalent, bivalent or tetravalent VLPs complexed with alum (alhydrogel 2%) as an adjuvant. The protein content of the individual VLP preps and the multivalent formulation was comparable (Supplementary Figure 3B**)**. Two immunizations were administered at week 0 and week 2 and the serum samples were collected at ~6 weeks post primary immunization **(**Supplementary Fig. 3C**)**.

**Figure 6:**
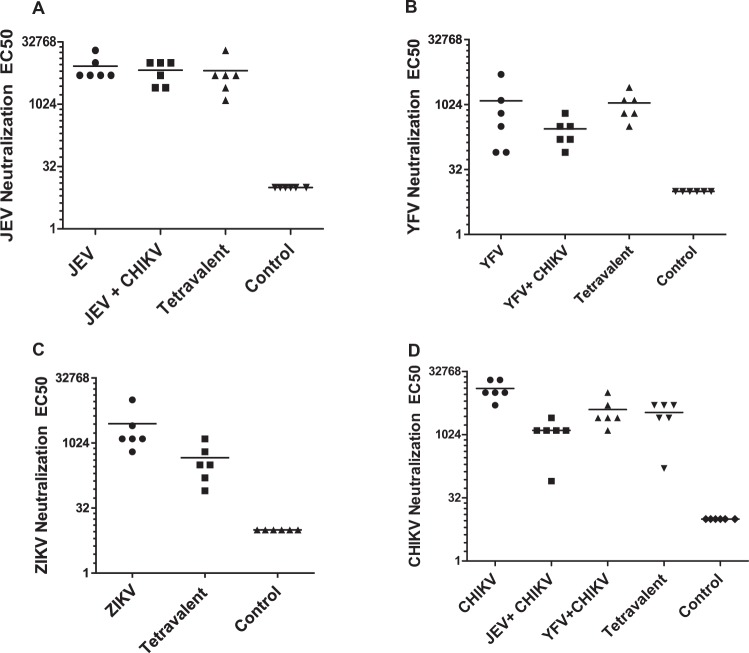
Neutralization efficacy of VLP vaccine combinations. Sera obtained from mice immunized with different VLP vaccine combinations were analyzed generation of neutralizing antibody response. Reporter virus based assays were used to determine the efficacy of neutralizing antibodies, curves were fit and EC50 values determined. Data for **(A)** JEV, **(B)** YFV, **(C)** ZIKV and **(D)** CHIKV neutralization EC50 values from each mice are shown. All samples were analyzed in duplicates.

Sera from immunized mice was tested for generation of neutralizing antibodies using the RVP assay. As demonstrated in Fig. 6A–D, high levels of neutralizing antibodies were detected in all the groups of immunized mice. As anticipated, the titers were highest for the monovalent groups as they received the highest antigen dose. Mice immunized with bivalent and tetravalent vaccine combinations showed a slight reduction in neutralizing antibody titers likely due to the reduction in antigen dose. These data demonstrate the feasibility of a multivalent arboviral vaccine in eliciting a strong neutralizing antibody response.

### Characteristic of immune response generated after immunization of mice with the above vaccine combinations

**Figure 7:**
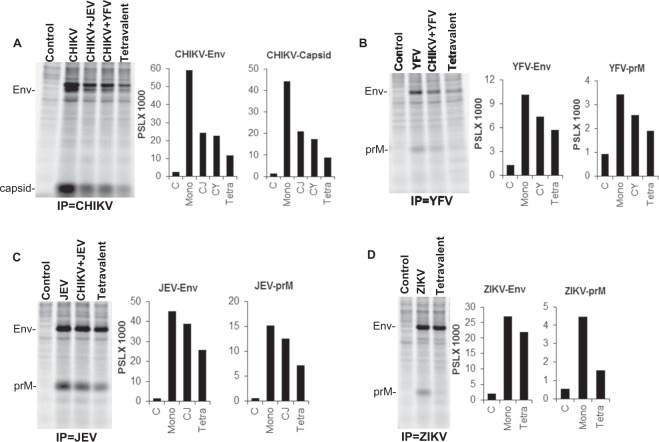
Quality of antibody response generated in VLP immunized mice. **(A)** CHIKV-E3-E2-E1 stable cell line was radiolabeled with [35S]Met-Cyst and cell lysates immunoprecipitated with pooled sera from indicated groups of immunized mice. Complexes were resolved on an SDS-PAGE gel followed by fluorography. **(B)** YFV-CprME stable cell line was radiolabeled with [35S]Met-Cyst and cell lysates immunoprecipitated with pooled sera from relevant groups of immunized mice. **(C)** JEV-CprME stable cell line was radiolabeled with [35S]Met-Cyst and cell lysates immunoprecipitated with pooled sera from indicated groups of immunized mice. **(D)** ZIKV-CprME stable cell line was radiolabeled with [35S]Met-Cyst and cell lysates immunoprecipitated with pooled sera from indicated groups of immunized mice. Quantitation of bands for Env, prM and Capsid protein is depicted in the graphs alongside each gel. Gel images were analyzed using the Multi Gauge software version 3.0 (http://www.winsite.com/fujifilm/fujifilm+multigauge/).

Our data in Fig. 6 clearly demonstrates that immunization of mice with monovalent, bivalent and tetravalent CJaYZ vaccine induces a potent neutralizing antibody response. We next looked at the characteristic of specific antibodies generated in immunized mice. For this, cell lysates form ZIKV, JEV, YFV and CHIKV VLP secreting stable cell lines were immunoprecipitated with pooled sera derived from different groups of immunized mice. As demonstrated in Fig. 7A and consistent with the neutralizing antibody data, the CHIKV monovalent VLP vaccine generated the strongest antibody titers followed by the bivalent and tetravalent combinations. Interestingly, for CHIKV, generation of Capsid antibodies was highly robust and also followed a declining pattern in the bivalent and tetravalent combinations. This clearly demonstrates the presence of Capsid protein in CHIKV VLPs and generation of an anti-Capsid immune response upon immunization. For YFV, JEV and ZIKV **(**Fig. 7B–D**)** again, specific E and prM antibodies were detected maximally in the monovalent vaccine with declining levels in the bivalent and tetravalent combinations. Overall, these data demonstrate the generation of specific YFV, JEV ZIKV and CHIKV E, prM and Capsid antibodies upon immunization with the multivalent VLP vaccine.

### Adaptation of cell lines to suspension growth and in serum free media for rapidly scalable VLP production

**Figure 8:**
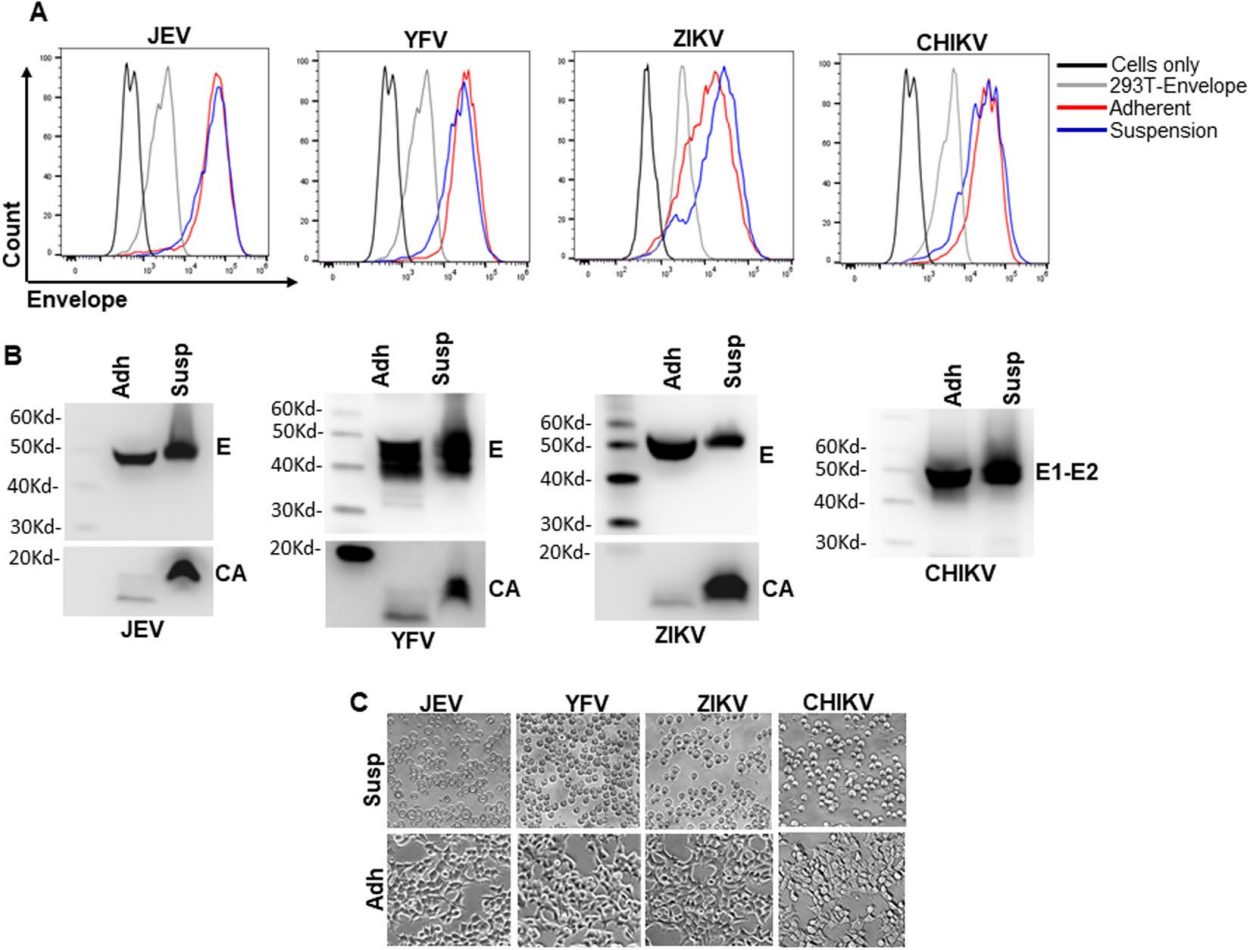
Adaptation of stable cell lines for growth in suspension culture. **(A)** JEV, YFV, ZIKV and CHIKV stable cell lines were adapted to grow in suspension culture. After complete adaptation, cells were stained for E protein expression via flow cytometry. The respective adherent counterparts were used as staining controls alongside. **(B)** VLP production from the adapted suspension cell lines was determined by western blotting for the E protein and compared to VLP secretion from the respective adherent counterparts. **(C)** Morphology of suspension cell lines via brightfield microscopy and comparison with their respective adherent counterparts. Flow cytometry histograms were created using the FloJo Software version 10.6.0 (Tree Star, https://www.flowjo.com/solutions/flowjo/downloads). Gel images were analyzed using GENETOOLS gel analysis Software version 4.03 (f) (Syngene, https://www.syngene.com/software/genetools-automatic-image-analysis/). Bright field images were analyzed using NIS Elements AR software version 3.2 (Nikon, https://nis-elements-viewer.software.informer.com/3.2/).

Commercial success of VLP vaccines is predicated on culture of large volume of cell lines in bioreactors. Moreover, propagation of cells in serum free media facilities exclusion of unwanted proteins present in serum. Although 293 T based cell lines produced in our study produce copious amounts of VLPs, the adaptation of these cells to suspension growth would facilitate commercial success of the vaccine platform. 293 T cells are amenable to suspension adaptation using specialized media and specific culture conditions^24^. We adapted the cells to grow in suspension by culturing them in 125 ml Erlenmeyer flasks in EX CELL Serum free medium. Cells were passaged multiple times and tested for presence of E antigen by flow cytometry and VLP secretion by western blotting. As demonstrated in Fig. 8C, adaptation of cells to suspension growth altered the cell morphology to rounded and detached compared to their adherent counterparts. Staining for E protein expression on the cell surface was comparable between the adherent and suspension cells **(**Fig. 8A**)**. Interestingly, the secretion of VLPs for the suspension cells was found to be much higher in the supernatant when compared to the same volume of adherent cell supernatants **(**Fig. 8B**)**. This is likely due to the growth of cells to higher density in suspension culture compared to adherent cells. These data demonstrate the feasibility of stable cell lines to be adapted to suspension growth in the absence of serum proteins which secrete high concentrations of VLPs in the culture supernatants.

## Discussion

Arboviruses are an emerging threat to human population in tropical and subtropical regions posing an increasing pressure on the healthcare system^25^. The rise in arboviral outbreaks including CHIKV, JEV, YFV and more recently ZIKV is driven by multiple factors including increased urbanization, increased global travel and trade as well as global climate changes^26^. While the incidence of arboviruses is on the rise, there is a lack of specific treatment for these diseases. Thus, there is a combined effort on prevention of infection by controlling the mosquito and tick vector populations, reducing mosquito and tick bites, travel advisories etc. However, such measures largely rely on availability of ample resources and human compliance and hence have proven less effective^27^. On a similar note, the antigenic relatedness combined with overlapping clinical presentation between these viruses makes differential diagnosis and treatment difficult^28^ suggesting that a more comprehensive approach that targets multiple arboviruses simultaneously is warranted. A multivalent vaccine targeting multiple arboviruses that are prevalent in majority of tropical and subtropical regions of the world would be highly desirable.

Vaccination against arboviruses has been an effective strategy in limiting outbreaks. Currently a JEV and YFV vaccine is available in monovalent form^29^. Similarly a tetravalent vaccine for DENV (DENV1-4) has recently been approved for use in individuals previously exposed to Dengue^30^. All of these vaccines include a form of live attenuated virus (LAV) that makes the production and use of these vaccines on a large scale challenging. For example, in the case of JEV, the vaccination programs are geared towards specific regions where the diseases are endemic or in the case of an outbreak^10^. Furthermore, the safety issue of live viruses precludes vaccination of certain group of people including immunocompromised individuals and pregnant women. This is especially relevant in the case of ZIKV vaccination where the target population is largely going to be pregnant women.

Virus like particles provide a safe and effective alternative to traditional live virus based vaccines^23^. These particles provide several advantages over other vaccine platforms including 1) resemble native viruses thereby providing repetitive antigens on the surface of VLPs^31^. 2) Are replication incompetent making them safe to use in immunocompromised populations. 3) Ease as well as consistency of production using stable cell lines provides easy scale up and commercialization. A VLP vaccine against CHIKV has shown efficacy in animal models and has now progressed to human trials^22^. Similarly a ZIKV VLP vaccine has been tested in our lab in small animal model and shown to generate high titer neutralizing antibodies that protect against live virus challenge^14^. While a number of arboviral VLPs have been developed including some for JEV and DENV, most of these studies utilize prME expression and the particles lack Capsid protein^15^. The inclusion of Capsid protein in all of our VLP preps provides an additional antigen and better immunogenicity as evident from our Zika studies^13,14^. Furthermore, previous studies on arboviral VLPs including CHIKV have been conducted in monovalent forms and there is no study on whether a multivalent VLP vaccine against related arboviruses would provide effective immunity^22^. Our study was geared towards determining whether a multivalent VLP formulation encompassing CHIKV, JEV, YFV and ZIKV would provide effective neutralizing response against all four viruses. The use of multivalent VLPs from closely related viruses is not unprecedented and in fact, the current Human Papilloma Virus (HPV) VLP vaccine is a 9 valent vaccine against HPV types 6/11/16/18/31/33/45/52/58. Several studies have demonstrated the efficacy and cost effectiveness of this multivalent VLP vaccine^32–34^.

In previous studies, our group has developed a prME and CprME VLP vaccine against ZIKV and demonstrated the superiority of Capsid protein containing VLP vaccine (CprME) over VLPs without capsid (prME)^13^. We have also developed a strategy to generate Capsid protein containing VLPs for ZIKV using a bicistronic vector that expresses the structural proteins CprME as well as the Zika NS2B3 protease. This bicistronic vector helped us establish stable cell lines that are high secretors of Zika CprME VLPs^14^. In this study, we modified the bicistronic vector by replacing CprME region of ZIKV with that of YFV or JEV. Interestingly, ZIKV NS2B3 was effective at cleaving C from prME for both JEV and YFV allowing us to use the vector to establish stable cell lines. We used a self-inactivating lentiviral system with Blasticidin resistance gene to generate stable single cell clones of 293 T cells secreting VLPs for the above four viruses. Extensive characterization showed that the cell lines were stable, produced VLPs relatively consistently either in the presence or absence of Blasticidin and could be scaled up to higher density cultures like five layer multiflasks or suspension cells for VLP production.

Although use of multilayer flasks and other modified surface cell culture vessels are available for adherent cell expansion; the adaptation of adherent cells to suspension growth can be efficient strategy to scale up production to bioreactors. 293 T cells are amiable to adaptation to suspension growth using specialized media and culture conditions^24^. We were able to adapt all of our stable cell lines to grow in 293 T Ex Cell serum free media in shaker cultures with no reduction in VLP production compared to adherent cells. Furthermore, the use of serum free media provides an added advantage of eliminating animal origin products for commercial production of VLPs using these adapted cells in bioreactors.

Immunization studies in Balb/c mice showed that the VLPs (monovalent, bivalent or tetravalent) generated neutralizing antibodies as determined via RVP based assay. Further, confirmation of the neutralizing potential will need to be conducted using infectious virus. We also find a dose dependent response against the antigen as tetravalent formulations had 1/4^th^ the amount of each antigen (total protein) compared to the monovalent vaccine. The tetravalent vaccine hence generated a comparatively lower neutralizing antibody titer determined by RVP based neutralization assays and anti E antibodies determined via immunoprecipitation. These findings are important as this suggests that the amount of antigen/VLPs from each virus can be adjusted to achieve desired neutralizing efficacy. However, in order to achieve this, it will be important to determine the precise antigen amount of each VLP in the multivalent mix preferably as E antigen equivalents. The effectiveness of the VLPs in different combinations as bivalent or tetravalent combinations also shows the versatility of the system and the possibility of tailoring the vaccine to different geographical regions. Overall, our study provides a significant advancement both in terms of VLP vaccines as well as the strategy of using multivalent VLP vaccine candidates to target arboviruses. Further studies in appropriate animal models are needed to determine if the neutralizing antibody titers elicited upon immunization with the multivalent VLP vaccine are able to protect against a lethal virus challenge.

## Methods

### Cell lines and reagents

Experiments were conducted in BSL2 containment following appropriate safely protocols and guidelines. 293 T and Vero cells were obtained from ATCC and cultured in DMEM supplemented with 10% FBS. K562 cells were obtained from ATCC and cultured in RPMI media supplemented with 10% FBS. All transfections were performed using Turbofect reagent (Thermo Fisher) as per the manufacturer’s instructions. Antibodies for detection of Zika Envelope (E) protein (GTX133314) and Capsid protein (GTX133317), JEV E protein (GTX125867) and Capsid protein (GTX634153), YFV E protein (GTX134024) and Capsid protein (GTX134022) by western blotting were from GeneTex. For detection of ZIKV, YFV and JEV E protein by flow cytometry, the 4G2 antibody (MAB10216, Millipore) was used. For CHIKV E1-E2 protein detection by flow cytometry and western blotting, the 6A11 clone was used from Novus Biologicals. JEV, YFV and ZIKV specific sera were kindly provided by BEI resources and the CHIKV specific sera was kindly provided by Centers for Disease Control.

### Plasmids

The Zika CprME vector has been described previously^13^. The WNV replicon reporter plasmid Rep/GFP was kindly provided by Dr. Ted Pierson (NIAID)^21^. Plasmid containing the WNV NS2B3 accessory fusion protein expressing the active protease was a kind gift from Dr. Frank Scholle (NC State Univ.) pNL4-3 R-E- plasmid was kindly provided by the AIDS reagent program. JEV and YFV CprME regions were developed based on a consensus sequence generated using full length genomic sequences available from the Virus Pathogen Resource (ViPR) database. The consensus sequence was translated and the amino acid sequence of the CprME region was codon optimized and synthesized using gene synthesis (GenScript Inc.). The synthetic genes were subcloned into pcDNA3.1+ expression vector. A codon optimized Zika NS2B3 containing an HA Tag and IRES sequence was synthesized by GenScript Inc. using gene synthesis technology and has been described previously^14^. This construct was subcloned downstream of the CprME cassette in the lentiviral vector pLentiZikaCprME to generate pLentiZika CprME-IRES-NS2B3^14^. To generate YFV and JEV bicistronic constructs, the Zika CprME in the pLentiZikaCprME vector was replaced with YFV and JEV CprME respectively. The NS2B-3 open reading frame was also cloned into pcDNA3.1+ vector using the directional pcDNA3.1 TOPO cloning kit (Invitrogen) to generate Zika-NS2B3.

A similar approach was taken to generate CHIKV structural protein (C-E3-E2-6K-E1) from a consensus sequence that was further codon optimized and gene synthesized. The CHIKV synthetic gene cassette was subcloned into pcDNA3.1+ vector as well as pLenti6/V5 vector (Invitrogen). The CHIKV E region (E3-E2-6K-E1) was PCR amplified from the synthetic gene construct and cloned into pCDNA3.1 TOPO vector to generate a CHIKV E expression vector.

### Reporter virus particle assay

Flaviviral Reporter Virus Particles (RVPs) were generated as described previously^13,35^. Briefly, 239 T cells were transfected with WNV Rep/GFP along with CprME plasmid from different flaviviruses (ZIKV, JEV, YFV). The RVPs were harvested 48 h post transfection, aliquoted and stored for future use. RVPs were titrated in Vero cells plated in 96 well clear bottom black plates at 5,000 cells per well. Thereafter, cells were infected with serial two-fold dilutions of the RVPs and incubated for 72 h. The plates were fixed using 4% formalin/PBS, images acquired using the Cytation5 imaging system (BioTek) which provides a read out of the number of GFP positive cells per well. For neutralization assays, mouse sera or antibodies were serially diluted in DMEM and incubated with the RVPs for 1 h at room temperature. Subsequently, the virus:sera mix was added to Vero cells and number of GFP positive cells quantitated as described above.

For the CHIKV reporter virus assay, HIV reporter particles pseudotyped with CHIKV E were used similar to the assay reported by Akahata *et al*.^22^. For this, the CHIKV Envelope region (E3-E2-6K-E1) was PCR amplified from the synthetic gene construct and cloned into pCDNA 3.1 TOPO vector to generate a CHIKV E expression vector. The HIV pNLLucR-E- vector that expresses the HIV genome minus the Env and includes a luciferase gene along with CHIKV Env was used to generate luciferase expressing CHIKV pseudotyped RVPs.

### Stable cell line generation

Methods for the generation of stable cell lines have been described previously^13,14^. Lentiviral vectors expressing ZIKV, JEV or YFV CprME-IRES-NS2B-3 were packaged into lentiviral particles in 293 T cells by transfecting with the lentiviral vector along with the helper construct php-dl-NA (NIH AIDS Reagent program) and VSVG Env. The viral supernatants were collected at 48 h post transfection, aliquoted and stored at −70 °C. To generate stable cell lines, 293 T cells were transduced with the lentiviral particles and the cells were selected using Blasticidin at a concentration of 10 μg/ml. Bulk selected cells were passaged 8–10 times and stained for ZIKV E protein expression using monoclonal antibody MAB10216 (4G2, Millipore) at regular intervals to confirm selection. Subsequently, single cell clones were generated from the bulk selected cells using limiting dilution cloning in 96 well plates. Each single cell clone was further characterized for E protein expression using immunostaining followed by flow cytometry. The release of VLPs from selected cell lines was determined by western blotting and the clone most potent at VLP release was selected for further studies. The same strategy was used for generation of CHIKV VLP secreting cell line. In this case the lentivirus expressed the CHIKV structural proteins (C-E3-E2-6K-E1). The transduced 293 T cells were bulk selected in the presence of Blasticidin before limiting dilution cloning followed by selection of single cell clones.

### Detection of viral E protein expression

Methods for the detection of viral E protein have been described previously^13,14^. This was conducted either via immunofluorescence microscopy, flow cytometry or western blotting. For fluorescence microscopy, the cells were fixed with 4% formaldehyde in PBS followed by permeabilization with 0.1% TritonX100/PBS. The cells were stained using E specific primary antibodies (4G2 or CHIKV E Ab; 1:200 dilution) followed by Alexa 488 conjugated goat anti-mouse IgG (Invitrogen, 1:200 dilution) and analyzed by fluorescence microscopy using the Nikon EclipseTi microscope. For flow cytometry analysis, cells were trypsinized, washed with PBS, fixed and permeabilized using the Cytofix Cytoperm reagent (BD Biosciences) as per the manufacturer’s instructions. Cells were stained using the primary antibody 4G2 or CHIKV E Ab at 1:500 dilution followed by secondary antibody Alexa 488 conjugated goat anti-mouse IgG (Invitrogen, 1:500 dilution). Cells were assayed by flow cytometry on a Gallios Flow Cytometer (Beckman Coulter) and at least 20,000 events for each sample were acquired. Data was analyzed using FlowJo software (Tree Star). For Western blotting, lysates were resolved on an SDS-PAGE gel, transferred onto PVDF membranes and probed with primary antibody (1:1000 dilution) followed by HRP conjugated secondary antibody and bands visualized via enhanced chemiluminescence using the super signal west femto maximum sensitivity substrate (Pierce). The protocol for radiolabeling of cells with [35S]Met/Cys protein labeling mix followed by immunoprecipitation of cell lysates has been described previously^35^.

### VLP production purification

VLPs for immunization were generated and purified from the selected stable cell lines for each virus as previously described^13,14^ with some modifications. Cell lines were seeded in 5 layer multi flasks (Corning) in DMEM supplemented with 10% FBS in the absence of blasticidin. Culture supernatants were harvested every 2–3 days at which point cells were sub-cultured. VLPs were concentrated as per the protocol of Brien *et al*.^36^. Harvested supernatants (25–30 ml) were transferred into ultracentrifuge tubes and carefully underlayered with 5 ml of 25% glycerol in TNE buffer. VLPs were pelleted by ultracentrifugation at 110,500 × g for 3 h at 4 °C. Thereafter, the supernatant was carefully removed and the VLP pellet resuspended in TNE buffer. The total protein content in the VLP preps was measured using the micro BCA kit (Pierce) and the presence of E protein in the preps was detected by western blotting using specific antibodies.

### Mouse immunizations

Animal studies were conducted as described previously^13,14^. All animal use was reviewed and approved by the TTUHSC El Paso Institutional Animal Care and Use Committee (IACUC). All experiments were performed in accordance with relevant guidelines and regulations. For immunization studies, 8 week old Balb/c mice were purchased from Jackson laboratory and housed in pathogen free animal facility at Texas Tech University Health Sciences Center, El Paso. Mice were divided into groups of six mice each and immunized with different monovalent, bivalent or tetravalent vaccine combinations conjugated with 2% Alhydrogel as adjuvant (InvivoGen). The VLP:Alhydrogel mix was injected intramuscularly in each thigh in a volume of 50 μl. Mice received one respective booster VLP dose at week 2 after the primary immunization. Control mice were sham injected with PBS mixed with Alhydrogel. Blood was collected at week ~7 post first immunization under terminal isoflurane anesthesia followed by intracardiac puncture. Blood samples were collected in serum separator tubes as per the manufacturer’s recommendations (Beckman Coulter). After coagulation, the tubes were centrifuged, sera harvested, aliquoted and stored at −80 °C until use.

### Adaptation of cells to suspension culture and serum free media

Stable cell lines secreting Zika, JEV, YFV and CHIKV VLPs were cultured in EX-CELL-293T serum free media (Sigma) supplemented with 6 mM L-glutamine, 25 mM HEPES buffer and antibiotics as per the manufacturer’s recommendations. Blasticidin was added at 5 µg/ml. Cells were transferred into vent cap 125 ml Erlenmeyer flasks and rotated in an orbital shaker at 110 rpm at 37 °C and 5% CO_2_. Cells were sub-cultured at regular intervals and re-seeded in appropriate numbers for further propagation.

## Supplementary information

Supplementary information.

## Data Availability

The datasets generated during and/or analyzed during the current study are available from the corresponding author on reasonable request.
