# Supplementary material for: Virus Like Particles (VLP) as multivalent vaccine candidate against Chikungunya, Japanese Encephalitis, Yellow Fever and Zika Virus
Source: Sci Rep. 2020 Mar 4;10:4017. doi: 10.1038/s41598-020-61103-1 (PMC7055223; doi:10.1038/s41598-020-61103-1)
Supplement: Supplementary file 1 — Supplementary information. [file 41598_2020_61103_MOESM1_ESM.pdf]

Multivalent Virus Like Particle (VLP) vaccine against Chikungunya, Japanese Encephalitis, Yellow Fever and Zika Virus.

Himanshu Garg*, Tugba Mehmetoglu-Gurbuz and Anjali Joshi *.

Center of Emphasis in Infectious Diseases, Department of Molecular and Translational Medicine, Texas Tech University Health Sciences Center, El Paso, TX, USA.

***Corresponding authors:**

Anjali Joshi and Himanshu Garg

Department of Molecular and Translational Medicine

Texas Tech University Health Sciences Center

5001 El Paso Dr, El Paso, TX, 79905

E mail: anjali.joshi@ttuhsc.edu; Himanshu.garg@ttuhsc.edu

Phone: 915-215-4263

Fax: 915-783-1253

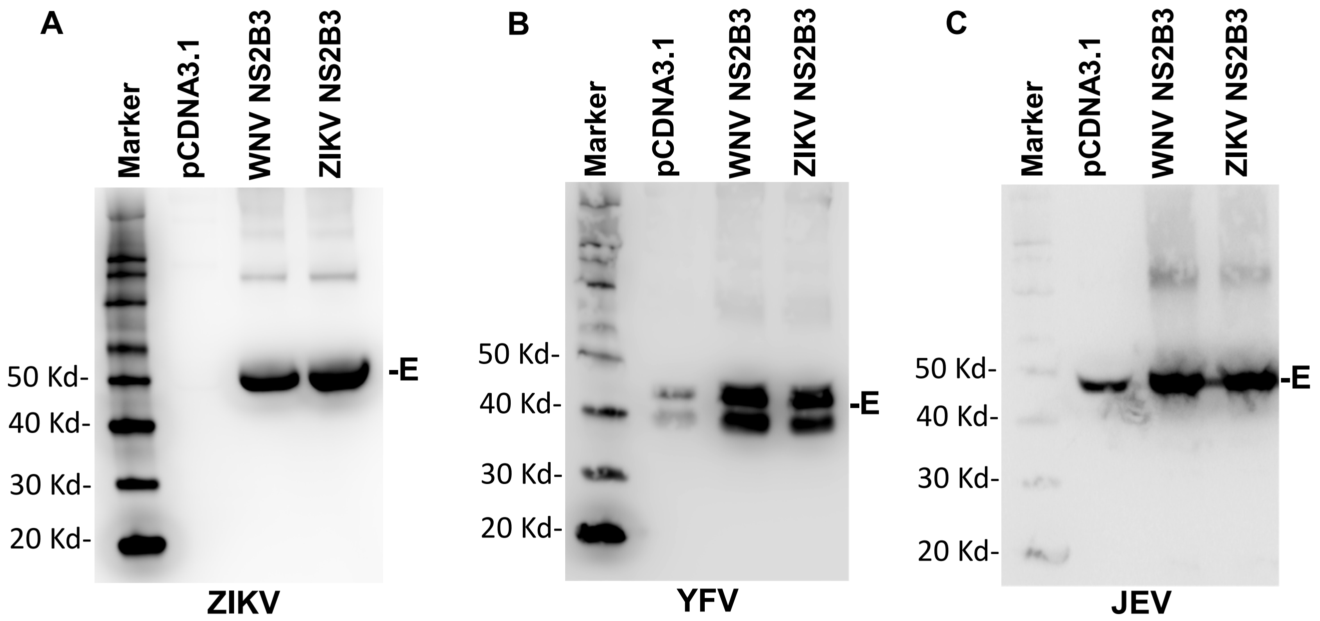

Supplementary Figure 1: Efficiency of ZIKV and WNV NS2B3 protease to cleave ZIKV, YFV and JEV CprME. 293T cells were transfected with control pCDNA3.1 vector or WNV or ZIKV NS2B3 expression vector. Culture supernatants were harvested 48h post transfection, concentrated and analyzed for **(A)** ZIKV **(B)** YFV or **(C)** JEV Envelope protein expression by western blotting. Gel images were analyzed using GENETOOLS gel analysis Software version 4.03 (f) (Syngene).

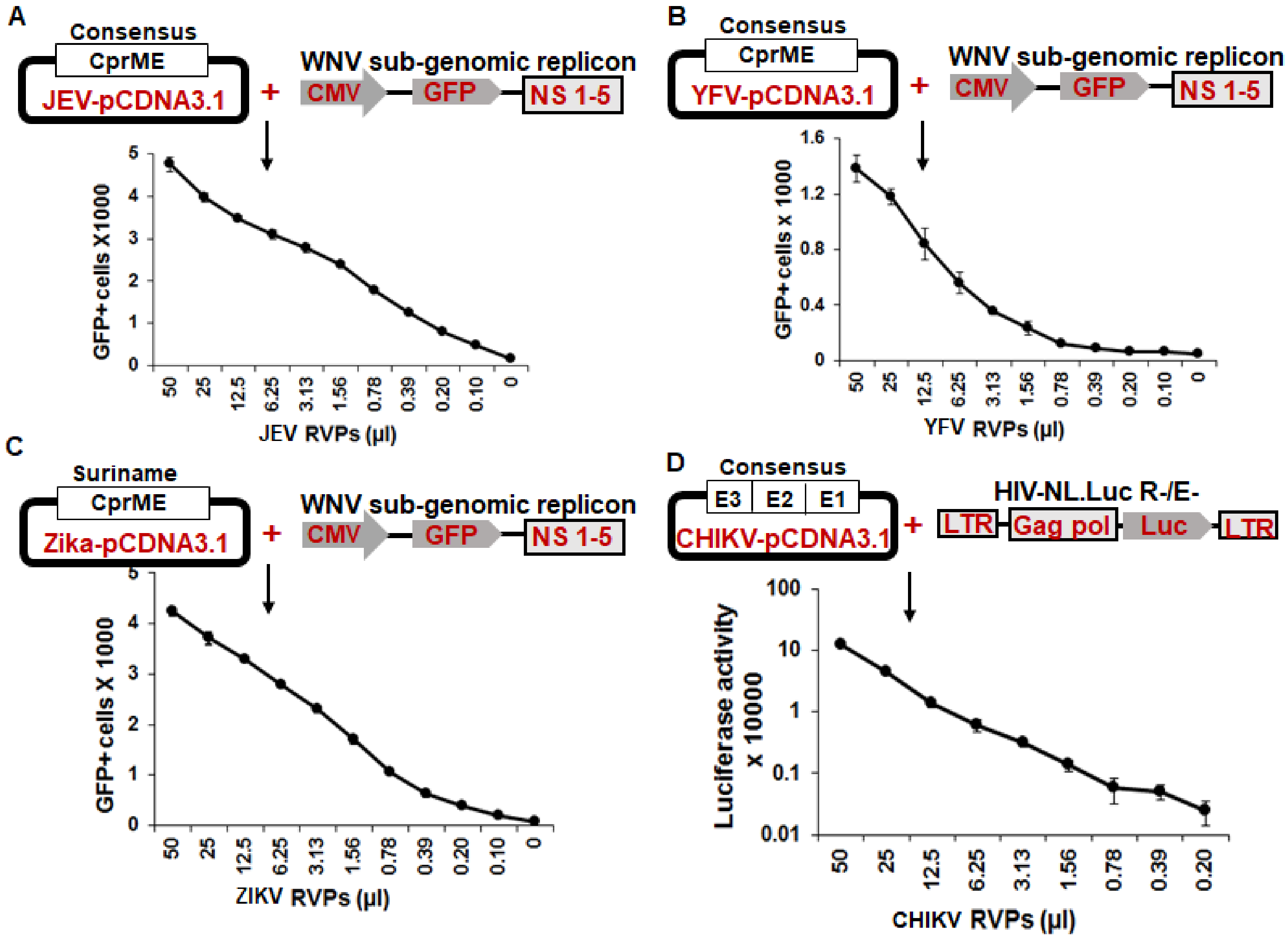

Supplementary Figure 2: Strategy for JEV, YFV, Zika and CHIKV RVP generation and titration. (A) 293T cells were transfected with JEV CprME expression vector along with the WNV sub-genomic replicon WNV Rep/GFP. Culture supernatants were harvested 48h post transfection and titrated in Vero cells using two fold dilutions of the virus stock. Number of GFP positive cells were determined 72hrs post infection using automated microscopy. **(B)** 293T cells were transfected with YFV CprME expression vector along with the WNV Rep/GFP. Culture supernatants were harvested and titrated in Vero cells as above. **(C)** 293T cells were transfected with Zika CprME expression vector along with WNV Rep/GFP. Culture supernatants were harvested and titrated in Vero cells as above. **(D)** 293T cells were transfected with the CHIKV E3-E2-E1 expression vector along with the HIV NL.Luc R-/E-. Culture supernatants were harvested and titrated in Vero cells as above. Data represent mean ± SD of triplicate observations.

A

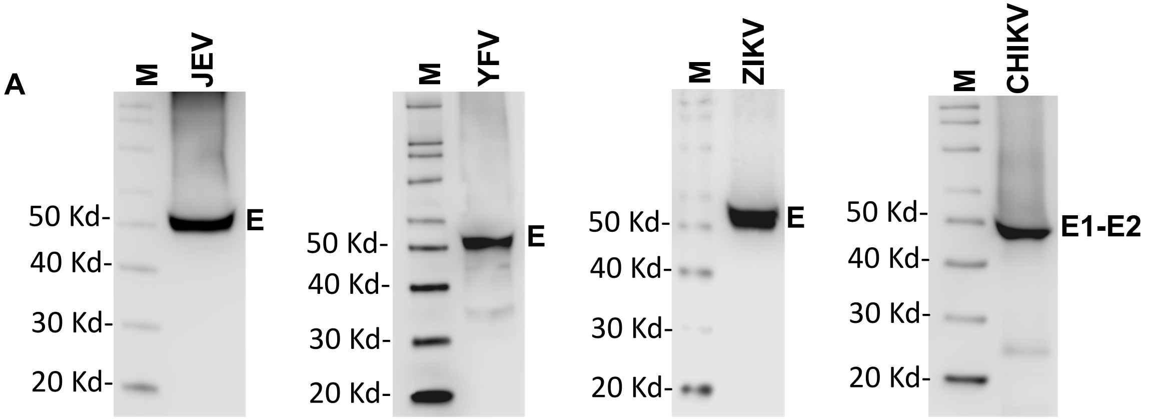

C

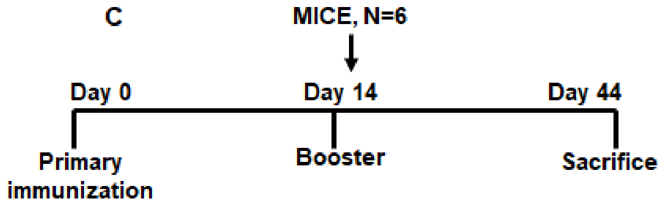

B

VLP	Protein µg/ml
JEV	1126.73
YFV	1260.19
CHIKV	959.91
ZIKV	1030.2
Tetravalent	1089.79

GROUP	INJECTION	ROUTE	DOSE (µg total protein)	AMOUNT
1	Zika	i.m.	100 µg	1:1 mix with Alum
2	JEV	i.m.	100 µg	1:1 mix with Alum
3	YFV	i.m.	100 µg	1:1 mix with Alum
4	CHIKV	i.m.	100 µg	1:1 mix with Alum
5	JEV+CHIKV	i.m.	50µg + 50µg	1:1 mix with Alum
6	YFV+CHIKV	i.m.	50µg + 50µg	1:1 mix with Alum
7	ZIKV+YFV+CHIKV+JEV	i.m.	25µg + 25µg + 25µg + 25µg	1:1 mix with Alum
8	PBS	i.m.	100 µl	1:1 mix with Alum

Supplementary Figure 3: VLP purification and schematic of mice immunization studies. (A) Single cell cultures producing JEV, YFV, ZIKV or CHIKV VLPs were propagated in 5 layer flasks. Culture supernatants were concentrated via ultracentrifugation, pooled and analyzed for E protein expression via western blotting. **(B)** Protein content of the individual VLP preps and multivalent formulation used for mice immunizations. **(C)** Balb/c mice were divided into 8 groups (N=6 mice/group) and immunized with the monovalent vaccine or different bivalent or tetravalent combinations as depicted in the table. Alum (2% alhydrogel) was used as adjuvant. Mice received a single booster dose at day 14 and were sacrificed at ~44 days post primary immunization. Gel images were analyzed using GENETOOLS gel analysis Software version 4.03 (f) (Syngene).

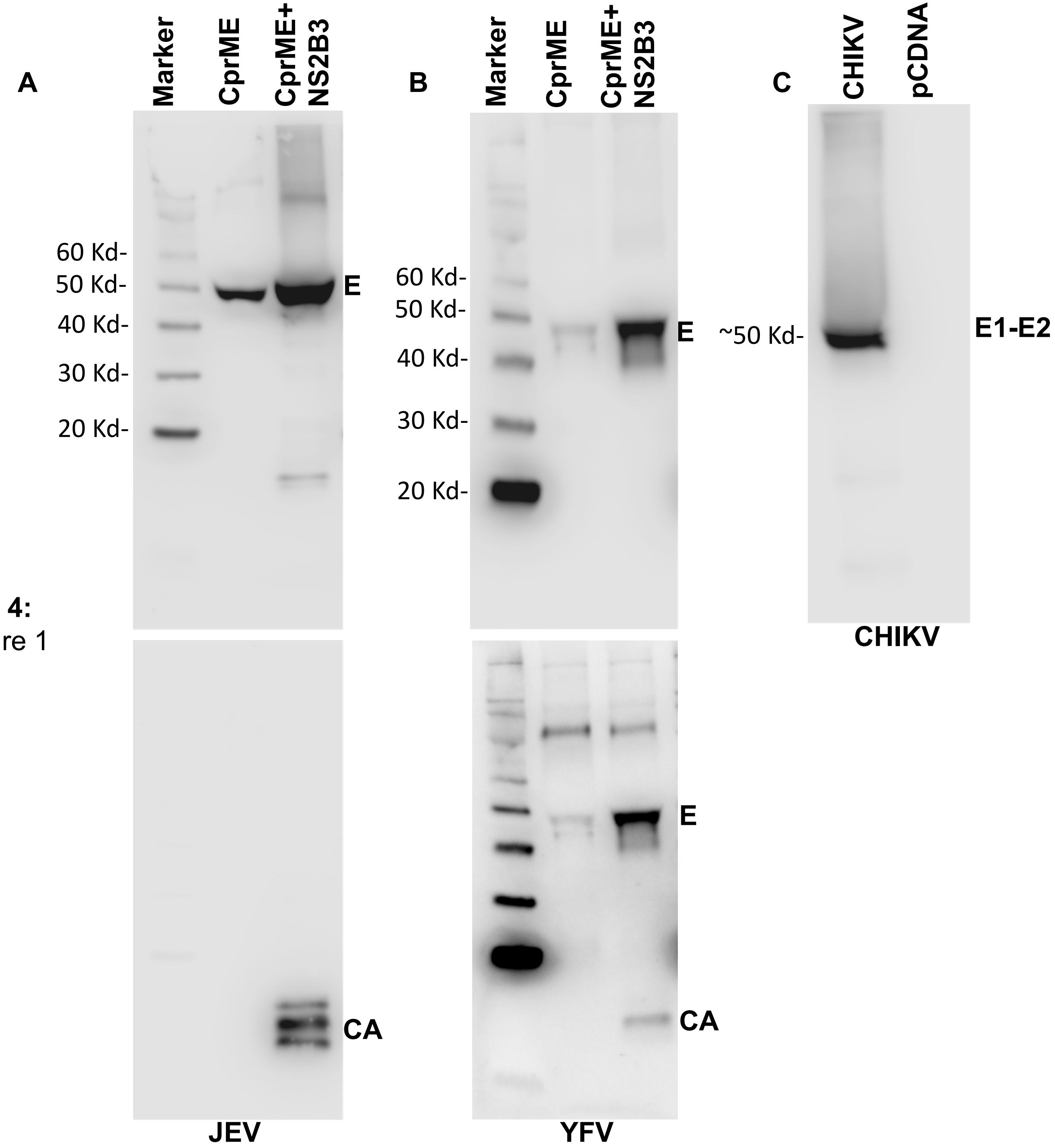

Supplementary Figure 4:
Uncropped gels for Figure 1

Supplementary Figure 5:
Uncropped gels for Figure 3

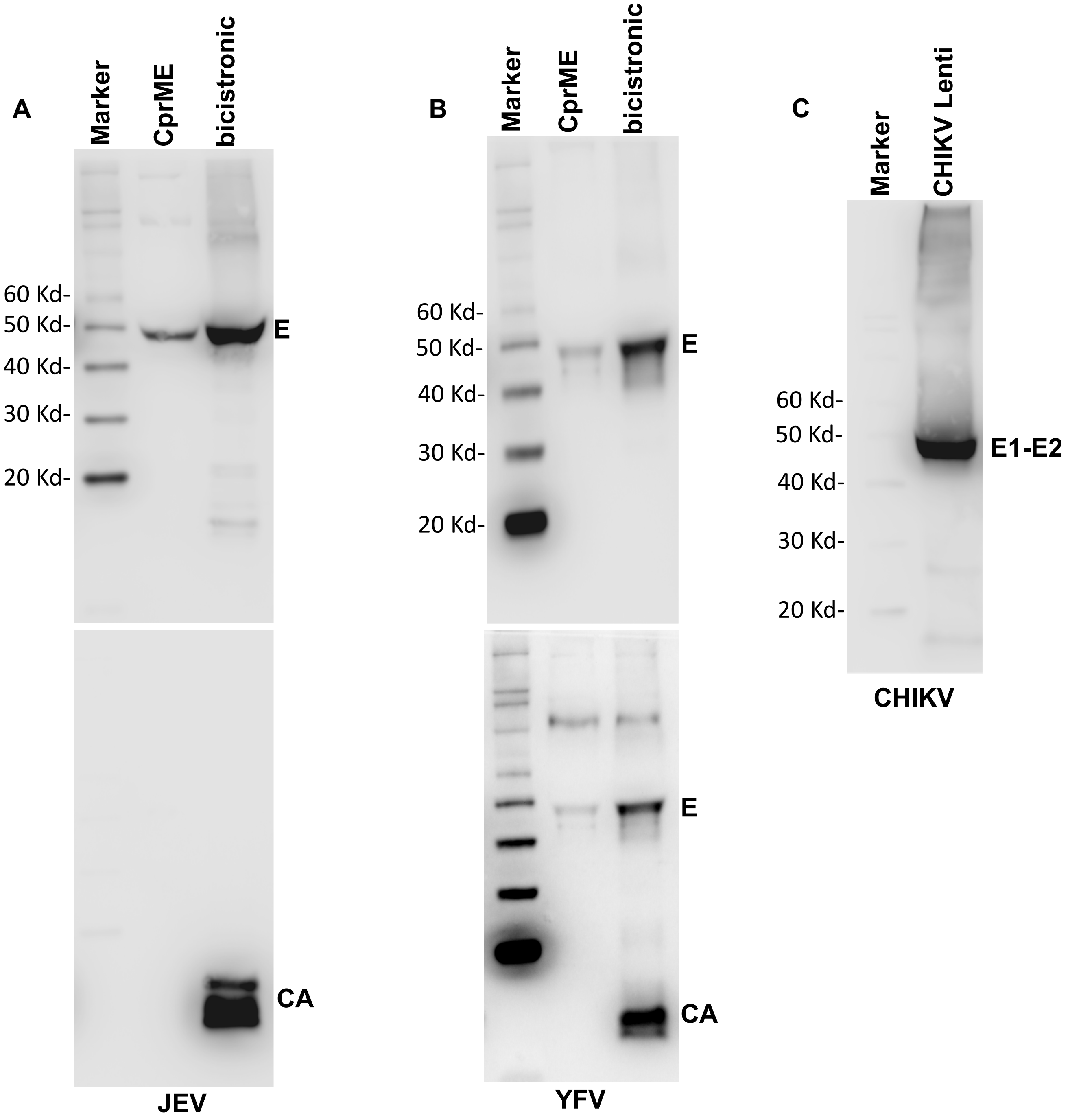

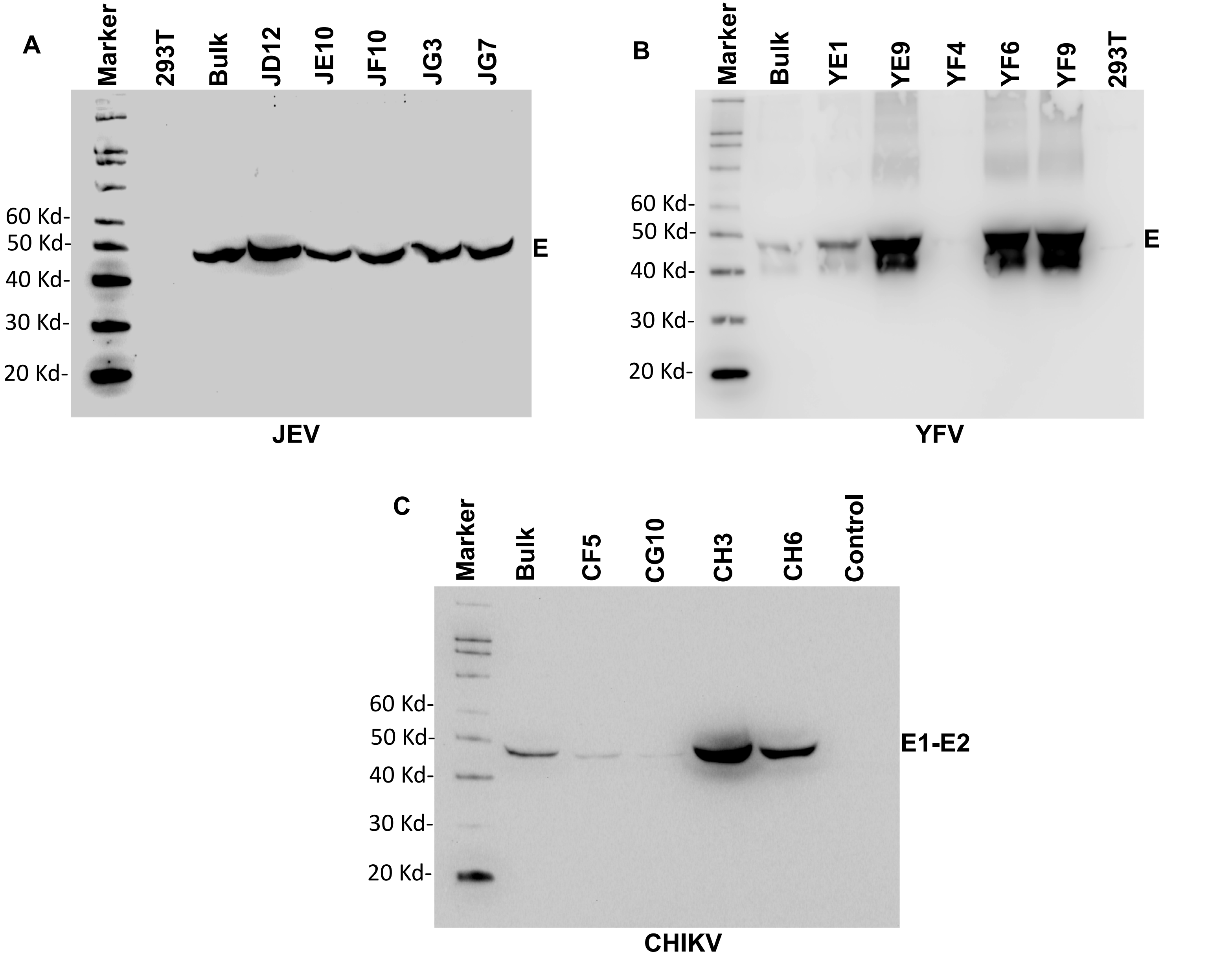

Supplementary Figure 6: Uncropped gels for Figure 4

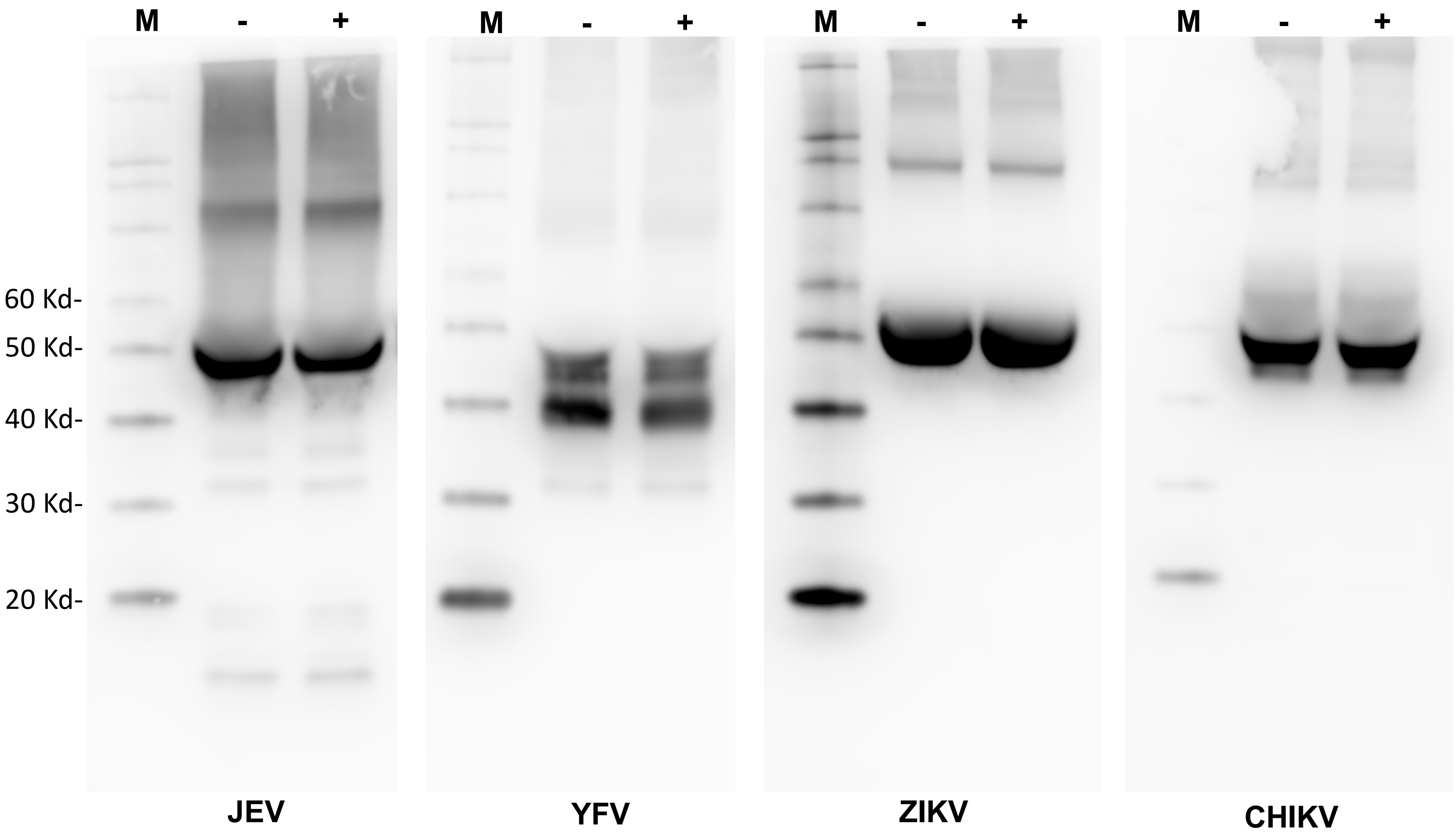

Supplementary Figure 7: Uncropped gels for Figure 5

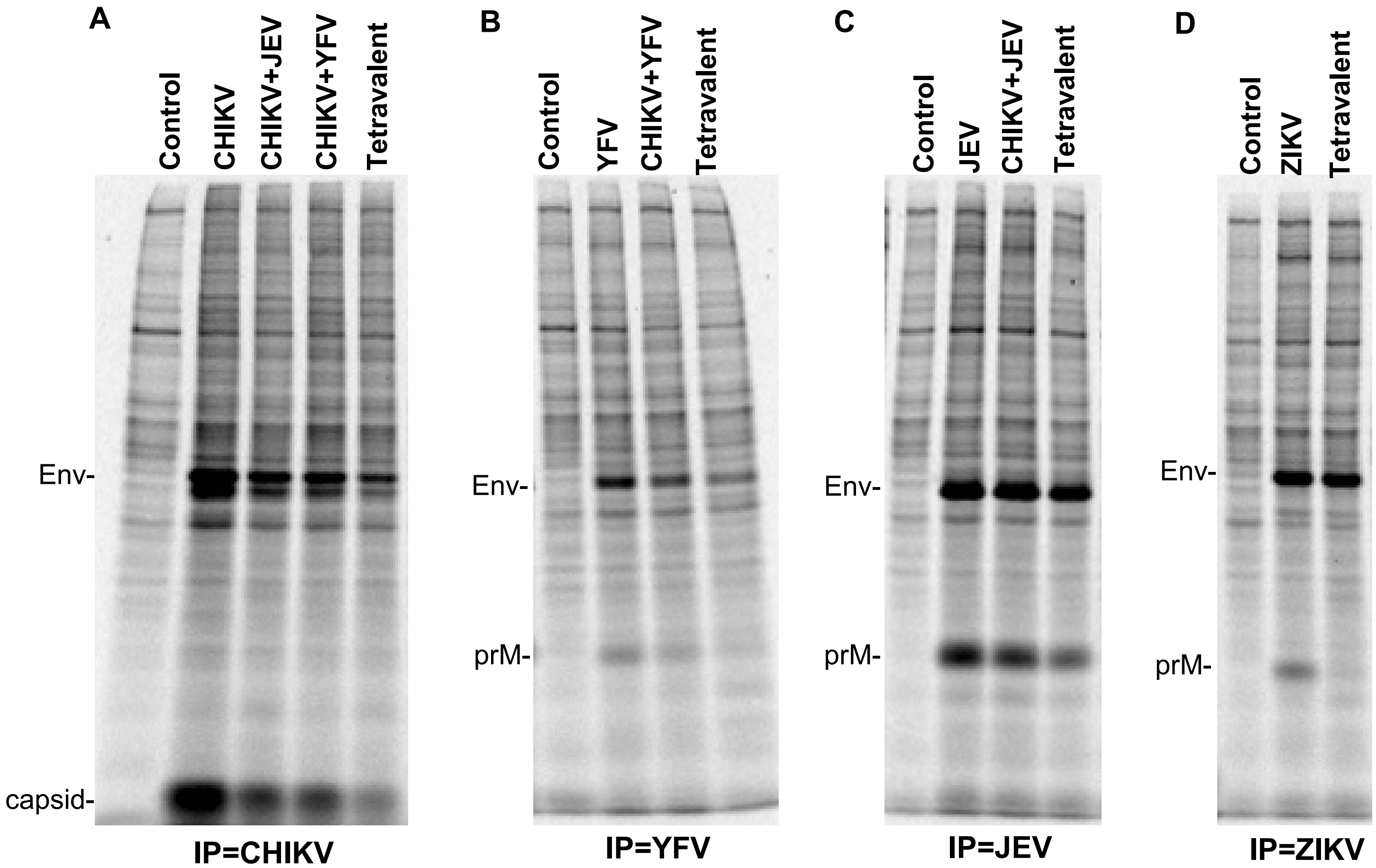

Supplementary Figure 8: Uncropped gels for Figure 7

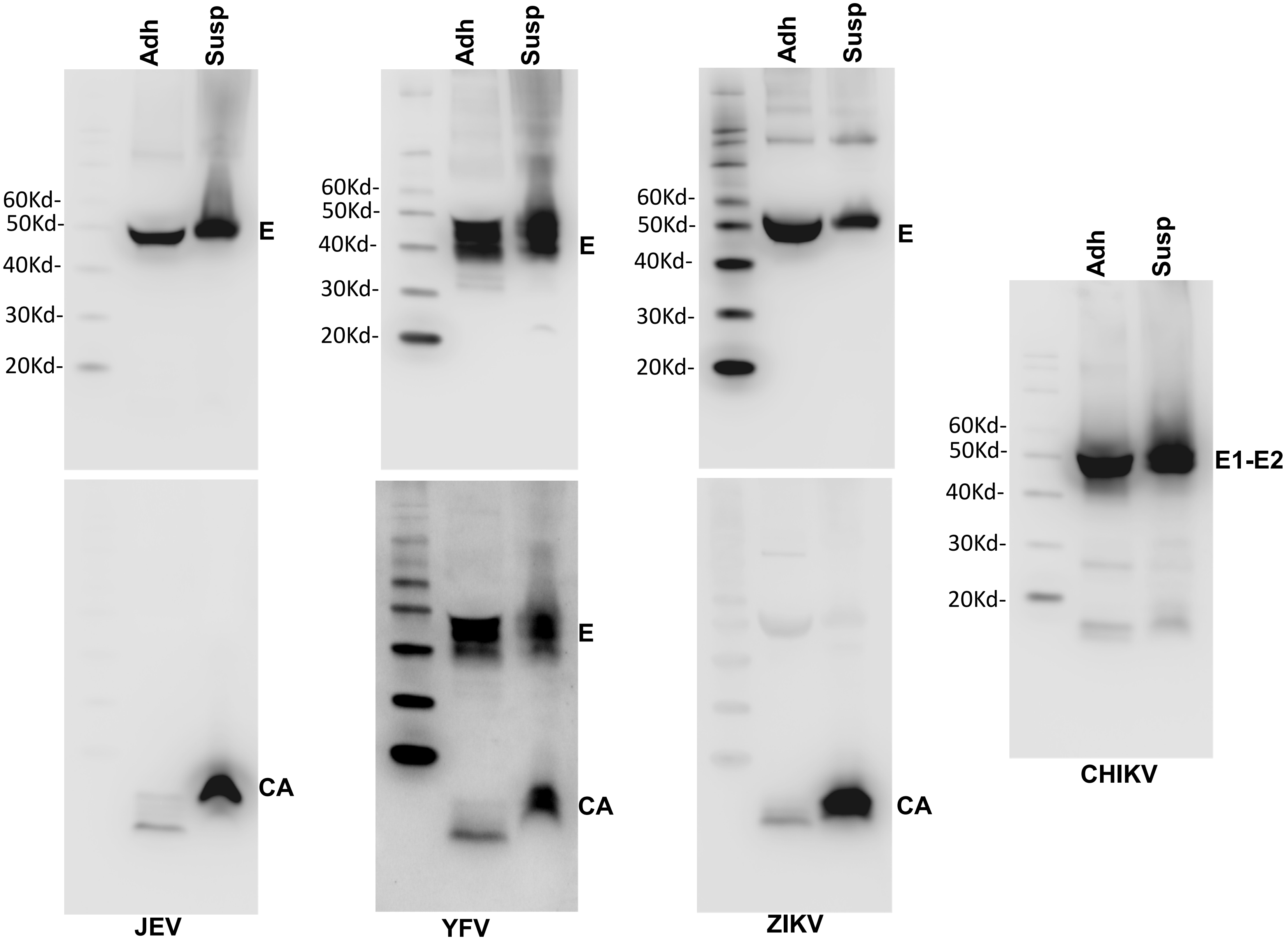

Supplementary Figure 9: Uncropped gels for Figure 8
